# A Phase I Clinical Trial of AAV2‐BDNF Gene Therapy for Alzheimer's Disease: Findings

**DOI:** 10.1002/alz70859_101305

**Published:** 2025-12-25

**Authors:** Mark H Tuszynski, Douglas W. Scharre, Gabriel C Leger, Russell R Lonser, Krystof S Bankiewicz, J. Bradley Elder

**Affiliations:** ^1^ University of California ‐ San Diego, La Jolla, CA USA; ^2^ Veteran Administration Medical Center, San Diego, CA USA; ^3^ Ohio State University Wexner Medical Center, Columbus, OH USA; ^4^ Ohio State University, Columbus, OH USA

## Abstract

**Background:**

BDNF is a neurotrophic factor that prevents neuronal death, activates neuronal function, restores synapses and improves learning and memory in animal models of Alzheimer's disease. Beneficial effects of BDNF gene delivery to entorhinal/hippocampal circuits on cognitive performance are observed in APP transgenic mice, aged rats and aged non‐human primates. Based on the preceding evidence, we advanced AAV2‐BDNF gene delivery to a Phase 1 clinical trial in patients with biomarker‐supported mild Alzheimer's disease (AD) and Mild Cognitive Impairment (MCI).

**Method:**

6 patients with mild AD and 6 patients with MCI are being recruited into this clinical trial. Patients undergo MRI‐guided injection of AAV2‐BDNF into the entorhinal cortex and are monitored over 2 years with serial cognitive testing and FDG PET scans. As of this writing, 6 patients with mild AD have been treated, and we are now beginning treatment of the MCI cohort.

**Result:**

6 patients with mild AD have been treated with followup times ranging from 1 to 18 months. There have been no serious adverse events related to the study procedure. Among three patients that were treated at least 6 months earlier, followup FDG PET scans demonstrate increases in cortical metabolism in entorhinal regions that received AAV2‐BDNF, a reversal of the normal pattern of decline typically observed in AD. Cognitive outcomes will be presented at the meeting. Given the safety of treatment to date, we are proceeding to dose the MCI cohort of patients.

**Conclusion:**

AAV2‐BDNF gene therapy to the entorhinal cortex in mild AD has been safe to date and restores FDG‐PET activity in the treated brain region. BDNF gene therapy offers the potential to prevent neuronal loss, activate neuronal function and restore synapses, representing a possible neuro‐restorative treatment for AD and MCI.

